# Cadmium-Induced Oxidative Stress and Apoptotic Changes in the Testis of Freshwater Crab, *Sinopotamon henanense*

**DOI:** 10.1371/journal.pone.0027853

**Published:** 2011-11-22

**Authors:** Lan Wang, Tuan Xu, Wen-wen Lei, Dong-mei Liu, Ying-jun Li, Rui-jing Xuan, Jing-jin Ma

**Affiliations:** School of Life Science, Shanxi University, Taiyuan, People's Republic of China; Florida International University, United States of America

## Abstract

Cadmium (Cd), one of the most toxic environmental and industrial pollutants, is known to exert gonadotoxic and spermiotoxic effects. In the present study, we examined the toxic effect of Cd on the testis of freshwater crab, Sinopotamon henanense. Crabs were exposed to different Cd concentrations (from 0 to 116.00 mg·L^−1^) for 7 d. Oxidative stress and apoptotic changes in the testes were detected. The activities of SOD, GPx and CAT initially increased and subsequently decreased with increasing Cd concentrations, which was accompanied with the increase in malondialdehyde (MDA) and H_2_O_2_ content in a concentration-dependent manner. Typical morphological characteristic and physiological changes of apoptosis were observed using a variety of methods (HE staining, AO/EB double fluorescent staining, Transmission Electron Microscope observation and DNA fragmentation analysis), and the activities of caspase-3 and caspase-9 were increased in a concentration-dependent manner after Cd exposure. These results led to the conclusion that Cd could induced oxidative damage as well as apoptosis in the testis, and the apoptotic processes may be mediated via mitochondria-dependent apoptosis pathway by regulating the activities of caspase-3 and caspase-9.

## Introduction

Cadmium (Cd), a toxic and nonessential element, is frequently used in electroplating, pigments, paints, welding, and batteries, which results in both biotic and abiotic environments [1]. Unlike organic compounds, Cd is not biodegradable and has a very long biological half-life [2]. Cd has been found to produce wide ranges of biochemical and physiological dysfunctions in humans and laboratory animals [3]. Many mammalian organs are adversely affected by Cd, which include kidney, liver, testis, lung, pancreas, prostate, ovary, and placenta [4]–[6], and several studies have illustrated that the testis is exceedingly sensitive to Cd toxicity [6], [7].

It is reported that spermatogenesis was disturbed by free radical. Mounting evidence has also shown that Cd alters antioxidant defense systems and increases production of cellular reactive oxygen species (ROS), such as singlet oxygen, hydrogen peroxide, and hydroxyl radicals [8]–[12]. ROS can lead to oxidative stress within cells by reacting with macromolecules and cause damages, such as mutations in DNA, destruction of protein function and structure, and peroxidation of lipids as well as alterations in gene expression and apoptosis [8]. Tissue levels of malondialdehyde (MDA) and the activities of superoxide dismutase (SOD), glutathione peroxidase (GPx) and catalase (CAT) are proven indicators of oxidative stress [9], [10]. Investigations by our lab have found that Cd changed antioxidant defense systems and induced apoptosis in the hepatopancreas of *S. henanense* [13], [14], suggesting that Cd-induced apoptosis may be connected with oxidative stress. Several reports have shown that oxidative stress is an important mechanism of Cd toxicity [15], [16].

In male experimental animals, Cd exposure can reduce testis weight and cause histopathological lesions leading to reduced sperm counts and impaired sperm motility and adversely affect male fertility [17]–[20], and several reports have shown that Cd can induce apoptosis in testis [21]–[23]. Many morphological and biochemical features have been used as criteria for confirmation of apoptosis [24]–[26]. Furthermore, Cd can directly activate endogenous endonuclease, resulting in cleavage of the DNA with the appearance of a characteristic ladder pattern [27]. In this process, caspase-9, the key enzyme, can cleave and activate downstream effector caspases, such as pro-caspase-3, and the active executioner caspases are responsible for cleaving of their target substrates to induce apoptosis [28].

Many studies concerning Cd-induced damages in testis have been performed on rodents [29], [30]. However, studies on Cd-injury in the testis of a crustacean are limited. *Sinopotamon henanense*, a representative of decapod crustacean, is widely distributed in freshwater areas of the Yangtze River drainage, Huaihe River drainage and Yellow River Valley of China. The background level of Cd was 0.019 µg/L [31] in the Yangtze River water system and the content of the Cd was 0.187–0.72 µg/g in the suspended particulate matters of the Yangtze River water system [32]–[35]. In some valleys near the Cd-rich mines, the Cd content in the sediments attained 231.7 mg/kg [34]. Furthermore, Cd contamination caused by human activity is much more prominent than by the natural erosion process in some places [36]. The dissolved Cd due to acidic pH resulted in the secondary pollution in aquatic environments. Freshwater crab lives in the sediments of streams and has the capability of accumulating heavy metals [37]. Therefore, the crab is a suitable bioindicator for aquatic heavy metals pollution [38]. Previous studies in our group have shown that distinct ultrastructural changes appeared in the testis after an injection of a dose of CdCl_2_ (1.5 µg/g) in *S. henanense* [39]. The present study was designed to investigate the cytotoxic effects of Cd on the testis and explore the probable mechanisms of apoptosis. The biochemical, physiological, and morphological alterations of the testis were investigated in *S. henanense* after acute Cd exposure.

## Results

### Effects of Cd on H_2_O_2_ contents in testes

**Figure 1:**
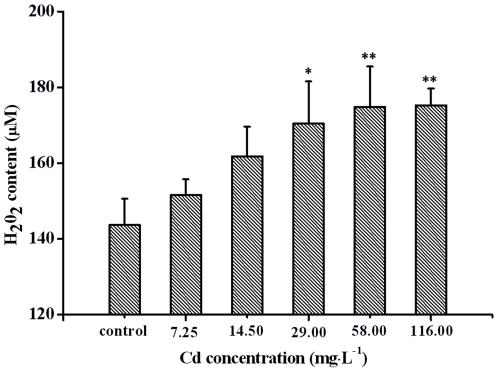
Effects of Cd on contents of H_2_O_2_ in the testis of *Sinopotamon henanense*. Data are expressed as mean±S.D.(n = 3). *P<0.05 vs. control group. **P<0.01 vs. control group.

There were no significant changes in H_2_O_2_ levels up to 14.50 mg·L^−1^, but a concentration-dependent increase in H_2_O_2_ contents was observed in crabs exposed to higher Cd concentrations. After exposure to 116.00 mg·L^−1^ Cd, H_2_O_2_ reached the highest level at a concentration of 175.21 µM, a 22% increase compared to the control (Fig. 1).

### Effects of Cd on MDA contents and SOD, GPx, CAT activities in testes

As shown in Table 1, the levels of MDA increased gradually in concentration-dependent manner and was positively correlated with the intensity of Cd stress. The maximal increase in MDA after 116.00 mg·L^−1^ Cd treatment was 178% greater than the control. Initially up to 14.50 mg·L^−1^ Cd resulted in a concentration-dependent increase in the activity of SOD, and a maximum increase of 55% was observed as compared to control. SOD activity showed a decrease in crabs exposed 29 mg·L^−1^ Cd but still above control levels. In the 116.00 mg·L^−1^ Cd treated group, a significant decline of SOD activity as compared to control was observed. Similar to SOD activity, the activities of GPx and CAT were also increased at first and then decreased when the Cd concentrations increased from 7.25 to 116.00 mg·L^−1^. The activity of GPx maximally increased 305% at 29.00 mg·L^−1^ of Cd intoxication, followed by a decrease of 54% in the group treated with the highest concentration of Cd (116.00 mg·L^−1^), and CAT activity was also dramatically increased 263% after exposure to 14.50 mg·L^−1^, and then dropped 16% in the 116.00 mg·L^−1^ Cd treated group compared with that of the control group.

**Table 1:** Effect of Cd on MDA levels and antioxidant enzyme activities in the testis of *S. henanense*.

Parameters	Concentration (mg·L^**−**1^)					
	0	7.25	14.50	29.00	58.00	116.00
MDA (nmol/mg protein)	0.51±0.16	0.89±0.36	1.06±0.13*	1.08±0.01*	1.15±0.35*	1.42±0.33**
SOD (U/mg protein)	4.16±1.05	4.58±1.73	6.47±0.47*	4.66±0.60	2.09±0.87*	1.40±0.69**
GPx (U/mg protein)	11.94±4.62	30.59±2.81**	37.08±1.50**	48.35±6.50**	20.76±6.01	5.53±1.73*
CAT (U/mg protein)	3.95±1.10	8.95±2.60*	14.32±3.19**	8.45±2.35*	5.55±1.35	3.30±0.52

Data are expressed as mean±S.D. (n = 3).

*P<0.05 vs. control group.

**P<0.01 vs. control group.

### Effects of Cd on light microscopy of testes

**Figure 2:**
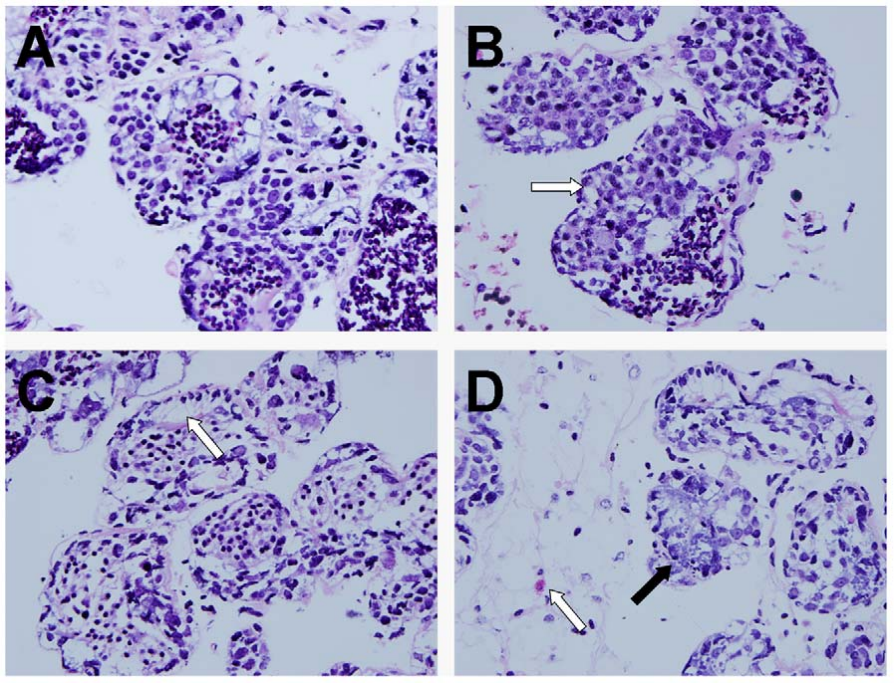
Effects of Cd on the testis of *Sinopotamon henanense* by Light microscope (400**×**). (A) Control group, the testis of control crabs showed normal structure, (B) Treated with 14.50 mg·L^−1^ Cd group, vacuolar degeneration appeared in the spermatogenic epithelium (white arrow), (C) Treated with 29.00 mg·L^−1^ Cd group, the number of sperms in the lumina of the seminiferous tubules was decreased (white arrow), (D) Treated with 116.00 mg·L^−1^ Cd group, the germinal layer in the seminiferous tubules exhibited extensive necrosis (black arrow) as well as edema in the interstitial space (white arrow).

The light microscopy examination of the testis of the control crabs showed normal structure as evidenced by well-organized distribution of cells in the seminiferous epithelium (Fig. 2A). At 14.50 mg·L^−1^ Cd, a large number of mature sperms were observed in the lumina of the seminiferous tubules, similar to the control, but vacuolar degeneration appeared in the spermatogenic epithelium (Fig. 2B). At the Cd concentration of 29.00 mg·L^−1^, the number of sperms in the lumina of the seminiferous tubules was decreased, and the seminiferous epithelia were atrophied as marked by a decrease in the number of germ cells (Fig. 2C). In the 116.00 mg·L^−1^ Cd treated group, the germinal layer in the seminiferous tubules exhibited extensive necrosis (Fig. 2D), associated with impaired spermatogenesis as well as edema in the interstitial space, and there were no spermatozoids in the lumen of many tubules.

### Morphological observation with fluorescence microscope

**Figure 3:**
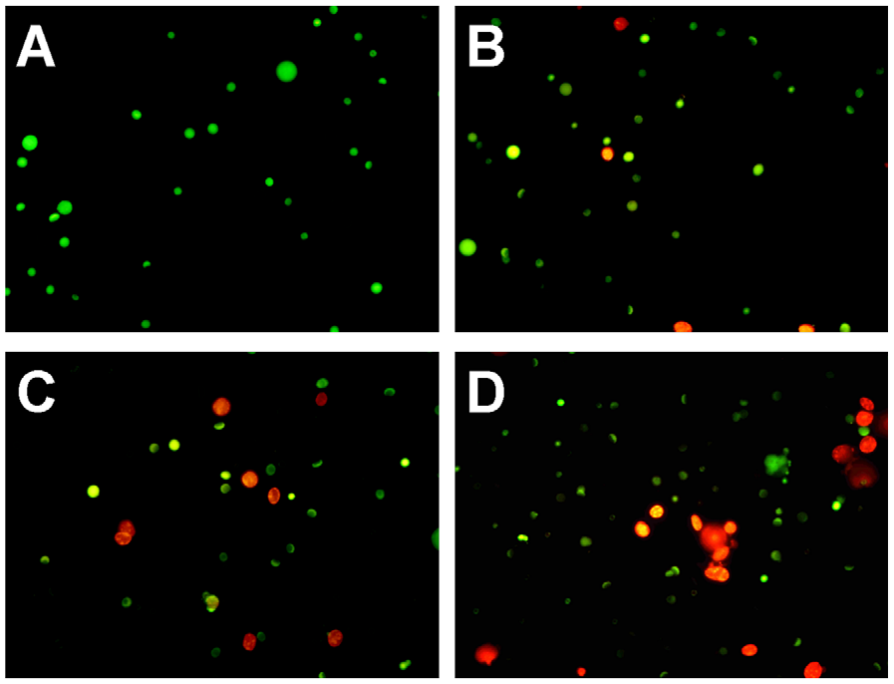
Effects of Cd on the testis of *Sinopotamon henanense* by fluorescence microscope. (A) Control group, (B) Treated with 7.25 mg·L^−1^ Cd group, (C) Treated with 29.00 mg·L^−1^ Cd, (D) Treated with 116.00 mg·L^−1^ Cd. Three types of cells can be recognized: live cells (green), live apoptotic cells (yellow), and dead cells by necrosis (red). Compared with the control group, the number of apoptotic cells were significantly increased with increasing Cd concentrations.

The fluorescence microscopic analysis results are shown in Fig. 3, and three types of cells can be recognized under fluorescence microscope: live cells (green), live apoptotic cells (yellow), and dead cells by necrosis (red). Fig. 3A showed normal (live) cells (green stain). With increasing Cd concentrations, the number of apoptotic and necrotic cells was significantly increased (Fig. 3B–D). Fig. 3D showed late apoptotic cells (yellow stain) and necrotic cells (red stain) when crab were treated with 116.00 mg·L^−1^ Cd.

### Effect of Cd on electron microscopy of testes

**Figure 4:**
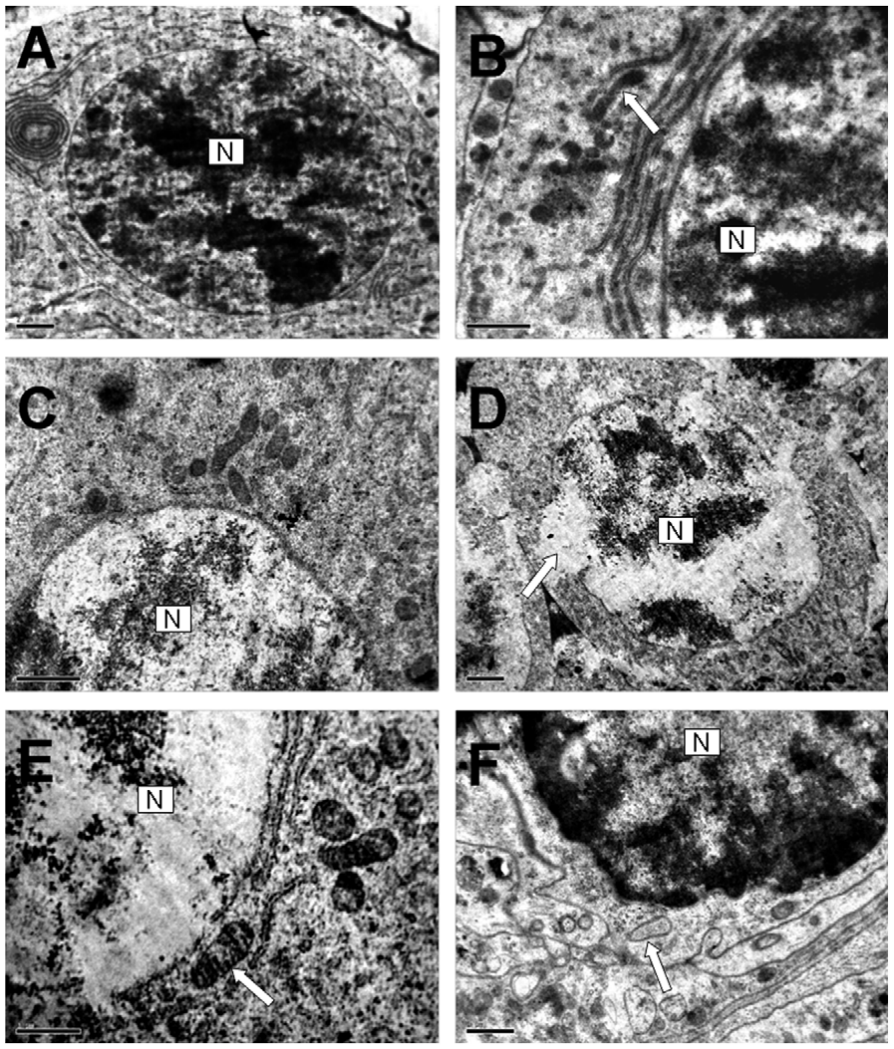
Effects of Cd on the ultrastructure of testis in *Sinopotamon henanense* by transmission electron microscope. (A) Control group, Nucleus (N) of spermatogomium was normal, Scale bar, 2 µm; (B) Control group, Primary spermatocyte mitochondria were normal with cristae (arrow), Scale bar, 1 µm; (C) Treated with 7.25 mg·L^−1^ Cd, no differences in the fine structure of secondary spermatocyte could be seen, Scale bar, 1 µm; (D) Treated with 29.00 mg·L^−1^ Cd group, the structure of the nuclear envelope partly disappeared in spermatogomium (arrow), Scale bar, 1 µm; (E) Treated with 29.00 mg·L^−1^ Cd group, the mitochondria underwent collapse of cristae in primary spermatocyte (arrow), Scale bar, 1 µm; (F) Treated with 116.00 mg·L^−1^ Cd group, the structure of nuclear envelope dispersed and finally disappeared, together with vacuolated mitochondria in primary spermatocytes (arrow), Scale bar, 1 µm.

The ultrastructural alterations were observed in testes of crabs treated with Cd. In control group, cytoplasm organelles and nucleus of spermatogomium and primary spermatocyte were normal, and mitochondria were normal with cristae (Fig. 4A–B). No differences in the fine structure of germ cells were seen in testes taken from controls and animals treated with 7.25 mg·L^−1^ Cd (Fig. 4C). When the Cd concentrations increased from 29 to 116.00 mg·L^−1^, the apoptotic germ cells were identified by electron microscopy, using morphological criteria of apoptosis. Characteristics for the apoptotic cells include condensation of nuclear chromatin and degeneration of cytoplasmic organelles. The structure of the nuclear envelope partly disappeared in spermatogomium (Fig. 4D) and the mitochondria underwent collapse of cristae in primary spermatocyte (Fig. 4E). During the late stages of apoptosis, the structure of the nuclear envelope was dispersed or outright missing, accompanied by chromatin condensation and marginalization in primary spermatocytes (Fig. 4F).

### Detection of apoptosis in testes by agarose gel electrophoresis

**Figure 5:**
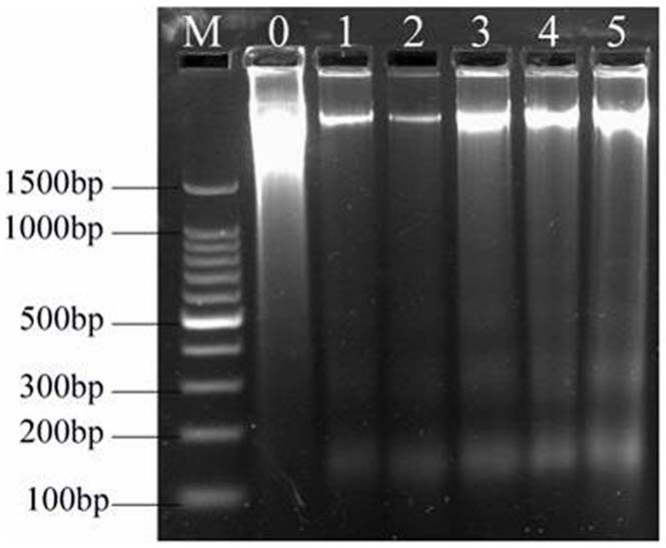
Internucleosomal DNA fragmentation in agarose gel electrophoresis induced by Cd (1.8%). Lane M: marker; 0, control group; 1, 7.25 mg·L^−1^; 2, 14.50 mg·L^−1^; 3, 29.00 mg·L^−1^; 4, 58.00 mg·L^−1^; 5, 116.00 mg·L^−1^.

The Cd-induced apoptotic DNA fragmentation in testis was clearly indicated on the agarose gel as detected by ethidium bromide fluorescence (Fig. 5). In the absence of Cd no ladder was observed (Fig. 5, Lane 0), and a genomic DNA ladder formation was observed when treatment of testes with Cd at 7.25 to 116.00 mg·L^−1^ (Fig. 5, Lane 1–5). The presence of Cd indicated a concentration-dependent, typical apoptotic fragmentation of the DNA. The degradation of DNA into oligonucleotide fragments was maximal at 116 mg·L^−1^ Cd, confirming the induction of apoptosis by Cd (Fig. 5, lane 5).

### Effects of Cd on Caspase-3 and -9 activities in testes

**Figure 6:**
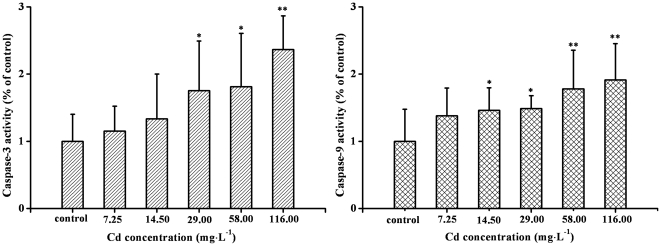
Effects of Cd on caspase-3, -9 activities in testes of *Sinopotamon henanense*. Each bar represents the mean of caspase-3, -9 activities for different experiments expressed as the percentage of enzyme activity found in control cultures. Asterisks indicate significant difference from control (*P<0.05; **P<0.01. n = 3).

The apoptotic process included the activation of cysteine proteases, which represent both initiators and executors of cell death. In this study, caspase-3 and caspase-9 activities were both increased in a concentration-dependent fashion after Cd treatment. As shown in Fig. 6A, 29 mg·L^−1^ Cd strongly induced the activativity of caspase-3 protease, and the maximum activity was about 2.4-fold of control at the highest concentration (116.00 mg·L^−1^ Cd). Similar to caspase-3 protease activity, the activity of caspase-9 protease was first detected in 14.5 mg·L^−1^ Cd, and the maximal caspase-9 activity was observed at 116.00 mg·L^−1^ Cd exposure, which was about a 1.9-fold of control (Fig. 6B).

## Discussion

Spermatogenesis is a complex multi-temporal process, including proliferation and differentiation of spermatogonia, meiosis and spermiogenesis. In this process, any of the affected areas are likely to cause spermatogenesis impairment, and even infertility.

Cd is an important heavy metal widely used in NiCd batteries, metal plating, pigments, plastics and alloys [40]. This metal stimulates free-radical production, resulting in oxidative deterioration of lipids, proteins and DNA, as well as initiating various pathological conditions in humans and animals [41]. Cd exerts adverse effects on structures and functions of reproductive organs directly at the testis level or by altering post-testis events such as sperm progress motility and/or function (viability), all of which may culminate in hypogonadism and infertility [3], [6], [17]–[19]. In the present study, we investigated the effects of Cd on oxidative stress and apoptosis of testes germ cells in crabs.

Oxidative stress, which is induced by reactive oxygen species (ROS) such as superoxide anion, hydrogen peroxide and hydroxyl radical, is known to play a critical role in testis injury [42]–[45]. Our investigation demonstrated that exposure to 58 or 116 mg·L^−1^ Cd significantly increased the content of H_2_O_2_ in testes compared to the control group, indicating that Cd induced the generation of reactive oxygen species (ROS). Similar to H_2_O_2_, MDA was also increased with increasing Cd concentrations, suggesting that the degree of damage to membrane lipids depended on the Cd concentrations. Furthermore, Our observations lend further support to the earlier study of Pathak et al. [9], [10], which demonstrated that one of the most damaging effects of these free radicals and their products in vivo was the peroxidation of membrane lipids. It is known that a loss in antioxidant capacities results in an intrinsic accumulation of MDA, which would be a reliable marker of free radical generation that indicates the risk of membrane damage [46].

The degree of cell damage under heavy metal stress depends on the rate of ROS formation and on the efficiency and capacity of detoxification and repair mechanisms. The cellular defense system against toxicity from ROS includes superoxide dismutase (SOD), catalase (CAT) and glutathion peroxidases (GPx). In present study, our results showed that low-concentration Cd stimulated the antioxidant activities probably due to the induction of an adaptive response, in the way of maintenance and/or increase of physiological activities under low concentration of Cd [47]. However, exposure to the high concentration (116.00 mg·L^−1^) of Cd was apparently linked to a significant reduction of antioxidant activities (SOD, CAT and GPx) compared to the control groups, indicating that the scavenging function of antioxidant activities were impaired under high concentration of Cd [48].

Oxidative injury also results in multiple physiological and pathological changes. In the present study, our investigation demonstrated that exposure to Cd induced histopathological changes of testis in a concentration-dependent manner. Our findings were consistent with the results of El-Ashmawy, Youssef [49] and Blanco et al. [48], which demonstrated that Cd induced disordered arrangement of germ cells, sloughing and a decreased spermatogenic cell layer in the seminiferous tubules, destruction of basement membranes, disintegration of spermatocytes, and complete absence of the sperms. Several studies implicated that Cd-related histopathological changes resulted from testis blood vessel damage, considered as the main cause of Cd toxicity [6], [50], as well as the mediator of the impaired testosterone secretion in mammalian testis [51]. The exact mechanism of Cd-induced histopathological changes in crustacean testis is unclear. However, this does not exclude the possibility of direct toxicity of Cd to germ cells [52].

Our findings support the results from other studies which indicated that Cd altered testis histology resulting in structural defects in germ cells [7]. Microscopic examination of AO/EB stained cells can be recommended as the most reliable method to distinguish viable, early or late apoptotic and necrotic cells [25]. In this study, three types of cells stained by AO/EB wererecognized under fluorescence microscope: live cells (green), live apoptotic cells (yellow), and dead cells by necrosis (red), indicating that the effect of Cd was related to the induction of apoptosis and/or necrosis of germ cells. The numbers of apoptotic and/or necrotic cells were increased with increasing Cd concentrations, in line with the results of histopathological changes in the present study. Another reliable method of detecting apoptosis is TEM, a powerful method to observe ultrastructural aspects. Our research is consistent with Edinger et al. [53], representing the typically apoptotic characteristics, including irreparablly cracked karyotheca, chromatin condensation, and changes in cytoplasmic organelle accompanied by vacuolation of the mitochondria. Wang et al. [54] indicated that mitochondrial vacuolation and swelling were caused by a high level of mutant SOD accumulation. Distinct ultrastructural changes supported the result of the histopathological assessment, and there was different extent of morphological damages in different germ cells, which may be dependent on the rate of Cd absorption and its distribution in the testes [55].

In addition to a series of morphological changes, the apoptosis program is concurrent with some changes in biochemistry [56]. Several studies suggest that Cd induces apoptosis, as indicated by the derivatives of Cd to stimulate DNA fragmentation [57]. In our study, testis exposure to Cd resulted in a DNA ladder confirming the induction of apoptosis, and this feature was clearly observed when crabs were treated with 58.00 and 116.00 mg·L^−1^ Cd. This result was similar to that of Lohmann et al. [27], reporting that Cd was able to stimulate calcium-dependent endonuclease in the Ca^2+^-free system, and the activation of the endonuclease responsible for apoptosis cuts the DNA into oligonucleotides in vitro in thymus cell nuclear.

Apoptosis is a tightly regulated physiological process [58], [59], although many potential stimuli can initiate apoptosis, it appears that these signals converge on the caspase pathway to execute the final phases of the apoptotic process [60]. In the present study, we found that caspase-3 and caspase-9 activities were both increased in a concentration-dependent manner after Cd treatment, suggesting that caspase-3 and caspase-9 were required for apoptosis induced by Cd in testes of crabs. The activities of caspase-3 were observed significant increased in testes of crabs exposed to 116.00 mg·L^−1^ Cd compared to the control group, and these results were in agreement with Ye et al. [61], indicating that apoptosis is characterized by a significant enhancement of caspase-3 activity. Caspase-9 located at the peak of caspase cascade activation, which is tarnally important for the activation of mitochondria-dependent apoptosis pathway [28]. In our study, we found a significant increase of caspase-9 activities in 58 and 116.00 mg·L^−1^ Cd compared to the control group, suggesting that mitochondria-dependent pathway was probably one of Cd-induced apoptotic mechanisms in testes of crabs.

In summary, the present study clearly demonstrated that acute exposure to Cd led to apoptosis in the testis cells of freshwater crab, which may lend strong support to the conclusion that acute exposure to Cd results in a cumulative and/or progressive testis injury. The possible mechanism of apoptosis induced by Cd in the testis of freshwater crab was mitochondria-dependent apoptosis pathway through activating caspase-9.

## Materials and Methods

### Reagent

All the chemicals used were of analytical grade, obtained from Sigma Co. (St. Louis, MO). MDA, SOD, GPx and CAT test Kits were purchased from Nanjing Jian Cheng Bioengineering Institute. Assay kits for Hydrogen peroxide, DNA extraction and caspase-3 and -9 activities were acquired from Beyotime Institute of Biotechnology (Jiangsu Province, China).

### Animals and treatments

Freshwater crabs, *S. henanense*, were purchased from the Dongan aquatic market of Taiyuan in China. Prior to experiments, crabs were acclimated in glass aquaria (45 cm×30 cm×30 cm) with 3–4 cm depth of dechlorinated tap water for 2 weeks, and the temperature was maintained at 20±2°C. Aquaria were shielded by a black plastic to reduce disturbance, and constant aeration was maintained. Water was changed three times a week and aquaria were cleaned thoroughly, and crabs were fed commercial fish feed. After acclimatization, healthy adult male crabs with a homogeneous size (carapace width 3.8–4.2 cm, weight 18–22 g) were selected and divided into six experimental groups including one control group and five Cd-treated groups (7.25, 14.50, 29.00, 58.00 and 116.00 mg·L^−1^). The acute exposure lasted for 7 days. During the experiment, crabs were not fed and dead animals were removed in time.

### Determination of hydrogen peroxide

Nine to eleven crabs from each group were randomly selected and the testes were immediately collected, weighed and then homogenized with 1∶9 (w/v) 0.86% saline solution at 4°C. The level of Hydrogen Peroxide (H_2_O_2_) was analysed by the method of Deiana et al. [62]. Briefly, test tubes containing 50 µl test solutions were placed at room temperature for 30 min and measured immediately with a spectrometer at a wavelength of 560 nm. The concentration of H_2_O_2_ released was calculated from standard concentration curve with triplicate experiments.

### Determination of MDA contents and SOD, GPx, CAT activities

Tissue samples of testes were immediately excised as described above. MDA content was determined by a method [63], based on reaction with thiobarbituric acid (TBA) at 90–100°C. SOD activity was measured by the Xanthine/xanthine oxidase method [64], one unit of SOD was defined as the amount of protein that inhibited the rate of NBT reduction by 50%. GPx activity was quantified by the dithio-binitrobenzoic acid method [65], based on the reaction between remaining glutathione after the action of GPx and 5,5′-dithio bis-(2-nitro benzoic acid) to form a complex that absorbs maximally at 412 nm. CAT activity was determined by the ammonium molybdate colorimetric method [66].

### Histological examination with light microscopy

Testes were removed quickly and then fixed for 24 h at room temperature by direct immersion in a 0.1 M pH 7.4 phosphate buffer with 4% paraformaldehyde. Samples were dehydrated with ethanol and toluene series and embedded in paraffin. Serial sections (4 µm) were mounted on gelatin-coated glass slides cut and stained with hematoxylin and eosin [67]. Slides were examined with a light microscope (Olympus BX51). Four sections were analyzed from each animal.

### Morphological analysis with fluorescence microscopy

Acridine orange/ethidium bromide (AO/EB) staining [68] of germ cells was performed to evaluate the cell death pattern induced by Cd. After 5 minutes of incubation, cells were pelleted and each sample was mixed with 1 µl of aqueous AO/EB solution ( 100 µg/ml of AO in PBS; 100 µg/ml EB in PBS ) just prior to fluorescence microscopy. Viable cells stained only by AO were bright green with intact structure; early apoptotic cells stained by AO were bright green area in the nucleus; Late apoptotic cells stained by AO low and EB were red-orange with condensation of chromatin as dense orange areas and reduced cell size seen in this study [25].

### Ultrastructure observation with transmission electron microscopy

Three to six small pieces of 1 mm^−3^ in size were taken from the testes and immersion-fixed in 2.5% glutaraldehyde in phosphate buffer (PH 7.4) immediately. Tissues were post-fixed in 1% osmium tetroxide and embedded in thin viscosity resin. Ultrathin sections were cut with an ultramicrotome (Leica UC-6), stained with uranyl acetate and lead citrate, and examined by transmission electron microscope (JEM-1400) and photographed.

### DNA fragmentation analysis

Fragmented DNA was isolated by DNA extraction kit (Beyotime, C0008) according to the manufacturer's instructions. The eluants containing DNA pellets were applied to electrophoresis on a 1.5% agarose gel at 80 V for 2 h. DNA bands were visualized and photographed by ultraviolet gel documentation system.

### Caspase-3, -9 activities assay

Tissue samples of testes were immediately excised as 2.3, and the caspase-3 and -9 activities were determined according to a modified method of Lasfer et al. [69]. Caspase-3 and -9 activities were measured through cleavage of a colorless substrate specific for caspase-3 (Ac-DEVD-ρNA) or caspase-9 (Ac-LEHD-ρNA) releasing the chromophore, ρ-nitroaniline (ρNA). An increase in absorbance at 405 nm was used to quantify the activation of caspases activities. After 7 days of exposure, testes were collected and lysed by a lysis buffer (40 µL) for 15 min on ice. Cell lysates were centrifuged at 18,000×g for 15 min at 4°C. Caspase-3, -9 activities in the supernatant were assayed using the kit. One unit is the amount of enzyme that will cleave 1.0 nmol of the colorimetric substrate pNA per hour at 37°C under saturated substrate concentrations.

### Protein determination

Protein concentration was determined using the Biuret method [70], with bovine serum albumin as a standard.

### Data analysis

Data were expressed as mean ± S.D., and assessed with one-way ANOVA followed by Fisher' least-significant difference (LSD) with SPSS 15.0 software. The difference between groups was considered to be significant at *P*<0.05.
