# Correction: Cadmium-Induced Oxidative Stress and Apoptotic Changes in the Testis of Freshwater Crab, *Sinopotamon henanense*

**DOI:** 10.1371/annotation/bae9fc08-fbfa-45b5-9d1d-0b8254d6efd5

**Published:** 2013-01-22

**Authors:** Lan Wang, Tuan Xu, Wen-wen Lei, Dong-mei Liu, Ying-jun Li, Rui-jing Xuan, Jing-jin Ma

The data in Table 1 in this article originate from the same experiment and overlap with the results in the last columns (day 7) in Figures 4, 1 ,2 and 3 from the authors' previous publication in Sichuan Journal of Zoology titled 'Effects of Cadmium on Spermatic Antioxidant Enzyme Activities and Lipid Peroxidation in Freshwater Crab Sinopotamon yangtsekiense'. This overlap as well as the publication in Sichuan Journal of Zoology should have been cited and described in the PLOS ONE article and the authors apologize for this omission. The publication in PLOS ONE focuses on cadmium-induced oxidative stress and apoptotic changes in the testis of freshwater crab Sinopotamon henanense, while the article published in Sichuan Journal of Zoology reported the effects of cadmium on antioxidant enzyme activities and lipid peroxidation in the same species. The PLOS ONE article reports original data on SOD, GPx, CAT activities and MDA content. The article in Sichuan Journal of Zoology incorrectly refers to the species employed as Sinopotamon yangtsekiense, however, the species used in the study is Sinopotamon henanense as reported in the PLOS ONE article. Based on its features and distribution, our group recently classified the species employed for the studies as S. henanense, instead of S. yangtsekiense as we had reported in previous publications. 

